# Reproduction of the Cancer Genome Atlas (TCGA) and Asian Cancer Research Group (ACRG) Gastric Cancer Molecular Classifications and Their Association with Clinicopathological Characteristics and Overall Survival in Moroccan Patients

**DOI:** 10.1155/2021/9980410

**Published:** 2021-07-28

**Authors:** Jean Paul Nshizirungu, Sanae Bennis, Ihsane Mellouki, Mohammed Sekal, Dafr-Allah Benajah, Nada Lahmidani, Hicham El Bouhaddouti, Karim Ibn Majdoub, Sidi Adil Ibrahimi, Sónia Pires Celeiro, Marta Viana-Pereira, Fernanda Franco Munari, Guilherme Gomes Ribeiro, Vinicius Duval, Iara Santana, Rui Manuel Reis

**Affiliations:** ^1^Biomedical and Translational Research Laboratory, Faculty of Medicine and Pharmacy, Sidi Mohamed Ben Abdellah University, Fez, Morocco; ^2^Department of Gastroenterology, Faculty of Medicine and Pharmacy, Abdelmalek Essaadi University, Tangier, Morocco; ^3^Laboratory of Epidemiology, Clinical Research and Public Health, Faculty of Medicine and Pharmacy, Sidi Mohamed Ben Abdellah University, Fez, Morocco; ^4^Department of Gastroenterology, Hassan II University Hospital, Fez, Morocco; ^5^Department of Visceral Surgery, Hassan II University Hospital, Fez, Morocco; ^6^Life and Health Sciences Research Institute (ICVS), School of Medicine, University of Minho, Braga, Portugal; ^7^ICVS/3B's - PT Government Associate Laboratory, Braga/Guimarães, Portugal; ^8^Molecular Oncology Research Center, Barretos Cancer Hospital, Barretos, São Paulo, Brazil; ^9^Pathology Department, Barretos Cancer Hospital, Barretos, São Paulo, Brazil

## Abstract

**Introduction:**

The Cancer Genome Atlas (TCGA) project and Asian Cancer Research Group (ACRG) recently categorized gastric cancer into molecular subtypes. Nevertheless, these classification systems require high cost and sophisticated molecular technologies, preventing their widespread use in the clinic. This study is aimed to generating molecular subtypes of gastric cancer using techniques available in routine diagnostic practice in a series of Moroccan gastric cancer patients. In addition, we assessed the associations between molecular subtypes, clinicopathological features, and prognosis.

**Methods:**

Ninety-seven gastric cancer cases were classified according to TCGA, ACRG, and integrated classifications using a panel of four molecular markers (EBV, MSI, E-cadherin, and p53). HER2 status and PD-L1 expression were also evaluated. These markers were analyzed using immunohistochemistry (E-cadherin, p53, HER2, and PD-L1), in situ hybridization (EBV and HER2 equivocal cases), and multiplex PCR (MSI).

**Results:**

Our results showed that the subtypes presented distinct clinicopathological features and prognosis. EBV-positive gastric cancers were found exclusively in male patients. The GS (TCGA classification), MSS/EMT (ACRG classification), and E-cadherin aberrant subtype (integrated classification) presented the Lauren diffuse histology enrichment and tended to be diagnosed at a younger age. The MSI subtype was associated with a better overall survival across all classifications (TCGA, ACRG, and integrated classification). The worst prognosis was observed in the EBV subtype (TCGA and integrated classification) and MSS/EMT subtype (ACRG classification). *Discussion/Conclusion*. We reported a reproducible and affordable gastric cancer subtyping algorithms that can reproduce the recently recognized TCGA, ACRG, and integrated gastric cancer classifications, using techniques available in routine diagnosis. These simplified classifications can be employed not only for molecular classification but also in predicting the prognosis of gastric cancer patients.

## 1. Introduction

Gastric cancer is the fifth most common cancer and the third leading cause of cancer-related deaths worldwide, being, therefore, a significant public health problem [1, 2]. According to the updated GLOBOCAN 2020 data, gastric cancer ranks 7th by incidence and 3rd by Morocco mortality [3]. Although recent advances in diagnosis and treatment, the clinical outcomes are often unpredictable, and they can vary widely among patients.

Understanding the molecular basis of gastric cancer pathogenesis is a critical phase to achieve personalized treatment of this disease. Several histological classification systems are used to define gastric cancer around the world. Lauren classification and the WHO classification (2010) are most commonly used, describing intestinal, diffuse, and mixed types in Lauren's classification and papillary, tubular, mucinous, and poorly cohesive types in WHO classification [4]. However, these classification systems have demonstrated little utility in clinical practice, as they do not have prognostic value and are without therapeutic implications.

The Cancer Genome Atlas (TCGA) network and the Asian Cancer Research Group (ACRG) have proposed novel classifications based on molecular profiling of gastric cancer. The TCGA study reported four major molecular subtypes: Epstein-Barr virus (EBV) positive tumors, microsatellite unstable (MSI) tumors, genomically stable (GS) tumors, and tumors with chromosomal instability (CIN) [5]. In 2015, the ACRG provided a new gastric cancer molecular classification, which also identified four molecular subtypes: MSI subtype, microsatellite stable with epithelial to mesenchymal transition features (MSS/EMT), MSS/TP53 mutant (MSS/TP53+), and MSS/TP53 wild-type (MSS/TP53-) [6]. Such molecular classifications have significantly expanded our insights into the heterogeneity and molecular complexity of gastric cancer. Despite this, high-throughput analysis technologies used in these studies are expensive and not available in routine practice.

Several studies have proposed simple classification systems of gastric cancer using immunohistochemistry (IHC) and EBV-encoded RNA in situ hybridization (EBER-ISH) as techniques available in most pathology laboratories around the globe [7–9]. In addition to the TCGA and ACRG classifications, another classification system that integrates both TCGA and ACRG subtypes, referred to as the integrated classification, was proposed [8, 10]. Although these studies have successfully defined gastric cancer molecular subtypes, their correlation with clinicopathological features and patient survival is still unclear.

In this study, we aimed to reproduce the results of TCGA, ACRG, and integrated classifications using routine diagnostic practice techniques in a series of 97 gastric cancer from North-East of Morocco. We also assessed the association between molecular subtypes, clinicopathological features, and patients' survival.

## 2. Materials and Methods

### 2.1. Patients

This study included 125 patients diagnosed with gastric adenocarcinoma at Hassan II University Hospital (Fez, Morocco) between January 2014 and December 2018. Patients with incomplete clinical data or insufficient formalin-fixed paraffin-embedded (FFPE) tumor tissue were excluded from the study (*n* = 28). Clinicopathological data were confidentially retrieved from medical records and anonymously inserted on an excel database.

### 2.2. Immunohistochemistry and In Situ Hybridization

Immunohistochemistry (IHC) was performed on FFPE tissue sections using different antibodies: polyclonal rabbit anti-human c-erbB-2 Oncoprotein (clone A0485, Dako; dilution 1 : 600), Ventana anti-E-cadherin (36) mouse monoclonal primary antibody (ready to use), flex monoclonal mouse anti-human p53 protein (clone DO-7, Dako; ready to use), and monoclonal mouse anti–PD-L1 antibody (clone 22C3; ready to use). The INFORM (Epstein-Barr virus Early RNA) probe was used to determine the EBV status by in situ hybridization (ISH). The INFORM EBER probe was detected with the ISH iView Blue Detection Kit on the Ventana BenchMark Ultra instrument.

Cases with known positivity for HER2, E-cadherin, p53, EBV, and PD-L1 were used as positive external controls. IHC results were interpreted as previously described [7, 8]. For p53 protein, complete loss or diffuse (≥80%) and strong nuclear staining were interpreted as p53 aberrant. E-cadherin expression was interpreted using a score of 0 to 3 (0= complete loss; 1= cytoplasmic expression; 2= both cytoplasmic and membranous expression; 3= membrane expression). Scores 0 and 1 were considered E-cadherin aberrant expression. Positive PD-L1 expression was defined as combined positive score (CPS) ≥ 1 [11].

CPS is the number of PD-L1 staining cells (tumor cells, lymphocytes, and macrophages) divided by the total number of viable tumor cells, multiplied by 100.

For EBER ISH, a case was considered EBV+ if the nucleus showed positive probe staining. HER2 immunoreactivity and gene amplification results were interpreted according to Hofmann's HER2 scoring system for gastric cancer [12]. Also, cases with equivocal HER2 IHC results (IHC score 2+) were assessed for gene amplification by fluorescence in situ hybridization (FISH). According to the manufacturer's instructions, FISH was conducted with the PathVysion HER2 DNA Probe Kit (Abbott Molecular).

### 2.3. Microsatellite Instability (MSI) Analysis

The MSI status of this series was determined by PCR multiplex in our recent study [13].

### 2.4. Rationale for Biomarker Evaluation

EBV-encoded small RNA (EBER) detection by in situ hybridization (EBER-ISH) is the gold standard for the evaluation of EBV-infected cells in tissue samples [14]. The MSI status of this series was previously determined using a multiplex PCR comprising five quasimonomorphic mononucleotide repeat markers (NR21, NR24, NR27, BAT25, and BAT26) [13]. This method allows accurate evaluation of tumor MSI status with 100% sensitivity and specificity [15]. While Bass et al. employed a series of multiple additional markers (ERBB2, CCNE1, KRAS, MYC, EGFR, CDK6, GATA4, GATA6, ZNF217, CD44, JAK2, CD274, PDCD1LG2,…) to distinguish the GS and CIN subtypes by the presence or absence of extensive somatic copy-number aberrations (SCNAs) [5], in this study, we distinguished the two subtypes by the E-cadherin immunostaining for the following reasons: (1) the TCGA study showed that GS tumors were enriched with diffuse histology according to the Lauren classification (73%), suggesting that the genetic features of GS tumors are associated with the diffuse phenotype [5]; (2) several studies have reported that aberrant E-cadherin expression was associated with diffuse histology in gastric adenocarcinoma [16, 17], and it has been suggested that loss of E-cadherin is a phenotypic expression of the genetic alteration noted in diffuse-type gastric adenocarcinoma (CDH1 mutations) [18]; and (3) adding other markers would impose a significant challenge in implementation of a subtyping algorithm in routine practice.

Several studies reported that the immunohistochemistry staining of p53 can be used as a robust method for inferring the presence of a TP53 mutation in cancer if the criteria of overexpression are stringently applied [19, 20], as in the present study.

### 2.5. Statistical Analysis

Statistical analyses were performed using SPSS v20.0 software (IBM SPSS Statistics, Chicago, IL, USA). Correlations between clinicopathological features and gastric cancer subtypes were analyzed using a chi-square test or Fisher exact test. Overall survival (OS) was estimated using the Kaplan-Meier method, and differences in survival within subtypes were examined using the log-rank test. The variables that were significant by univariate analysis were included in the multivariate analysis. The multivariate analysis was performed using the Cox proportional hazards regression model. A *p* value less than 0.05 was considered significant.

## 3. Results

### 3.1. Patient Clinicopathological Characteristics

Herein, we analyzed 97 gastric cancer patients, comprising 59 men (60.8%) and 38 women (39.2%). The mean age at diagnosis was 59.8 years (range: 28-85), and 25 (25.8%) patients were smokers. Three patients (3.1%) presented with a gastric ulcer history, and 7 (7.2%) presented with a gastrectomy history. Regarding the tumor location, 31 (32%) of the cases were cardia cancers, and 66 (68%) were noncardia cancers. Tumors were well or moderately differentiated in 62 (63.9%) cases and poorly differentiated in 35 (36.1%) cases. According to Lauren's classification, 65 (67.1%) of patients had intestinal tumors, and 32 (32.9%) had diffuse or mixed tumors.

### 3.2. TCGA Classification

**Figure 1:**
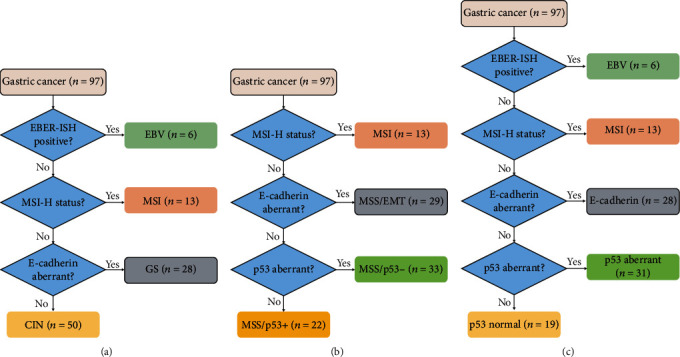
Flowchart illustrating how gastric cancers are divided into subgroups according to the (a) TCGA classification, (b) ACRG classification, and (c) integrated classification.

**Figure 2:**
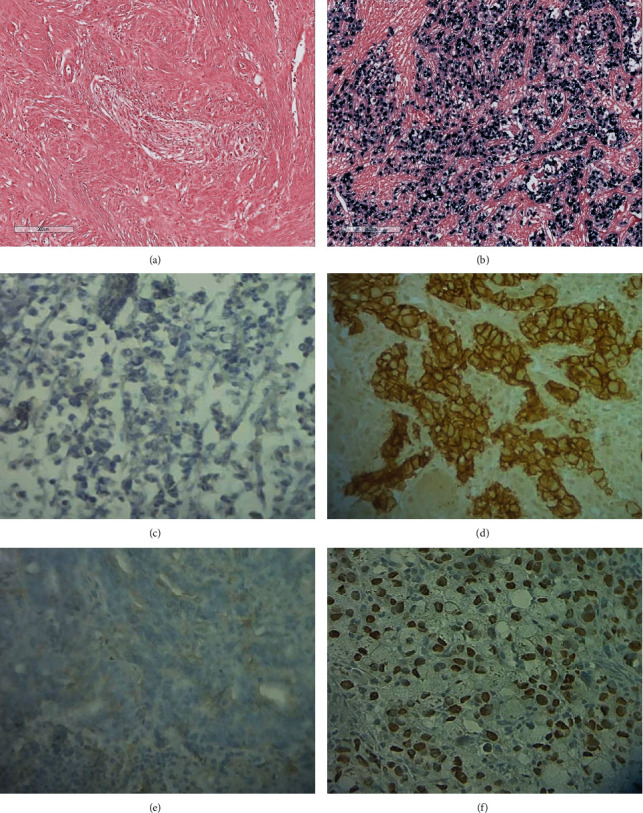
Immunohistochemical and EBER ISH analysis. (a) EBER negative, (b) EBER positive, (c) E-cadherin negative, (d) E-cadherin positive, (e) p53 negative, and (f) p53 positive (magnification ×400).

The TCGA study classified gastric cancer into four molecular subtypes: EBV, MSI, GS, and CIN [5]. To reproduce this classification, we used an algorithm based on the analysis of a panel of three markers: EBV, MSI, and E-cadherin (Figure 1(a)). Firstly, we identified the EBV subtype based on the EBER-ISH positivity. Then, all MSI-H tumors were classified into the MSI subtype. The remaining two subtypes were distinguished by E-cadherin immunostaining. Tumors with E-cadherin aberrant expression were classified into the GS subtype, and the remaining cases were categorized into the CIN subtype, as previously described [8, 10]. Out of 97 gastric cancer cases, 6 (6.2%) were EBV subtype (Figure 2(b)), 13 (13.4%) were MSI subtype, 28 (28.9%) were GS subtype, and 50 (51.5%) were CIN subtype.

The main clinicopathological characteristics of gastric cancer patients according to the TCGA subtypes are summarized in Table 1. EBV gastric cancers were observed exclusively in males, and 33% had a history of gastric ulcers.

In the MSI subtype, 69% of patients had the primary tumor located in a region other than the cardia (distal stomach). The MSI tumors showed a higher PD-L1 expression compared to EBV, GS, and CIN tumors. The GS tumors were diagnosed more frequently in younger patients (median age, 56 years) and exhibited predominantly diffuse histology (57%). Tumors with aberrant p53 expression (Figure 2(f)) were more frequent (62%) in the CIN subtype compared with other subtypes (33% in MSI, 38% in EBV, and 39% in GS subtype). This CIN subtype was characterized by an increased proportion of intestinal tumors (80%). Our data showed that 9/97 (9.3%) of patients were HER2+, and most of them were from the CIN subtype (5/9, 55.6%). Among intestinal tumors (*n* = 65), 40/65 (61.5%) were classified in the CIN subtype, followed by the MSI subtype (11/65; 16.9%), GS subtype (11/65; 16.9%), and EBV subtype (3/65; 9.2%). Among diffuse-mixed tumors (*n* = 32), 17/32 (53.1%) were categorized in the GS subtype, followed by the CIN subtype (10/32; 31.2%), EBV subtype (3/32; 9.4%), and MSI subtype (2/32; 6.2%).

### 3.3. ACRG Classification

The ACRG categorized gastric cancer into four clinically relevant molecular subtypes: MSI, MSS/EMT, MSS/p53-, and MSS/p53+ [6]. MSI, E-cadherin, and p53 were analyzed in 97 gastric cancers, and the ACRG classification was approximated using a simplified subtyping algorithm (Figure 1(b)). We first identified the MSI tumors. Of the MSS tumors, E-cadherin immunoexpression was used to identify the MSS/EMT tumors characterized by E-cadherin aberrant expression. Afterward, p53 immunostaining was used to classify the remaining cases into MSS/p53- (p53 aberrant expression) and MSS/p53+ (p53 normal expression). The results showed that MSI gastric cancer constituted 13.4% of the cases (*n* = 13); MSS/EMT gastric tumors represented 29.9% of the cases (*n* = 29); MSS/p53- tumors counted 34% of the cases (*n* = 33) and MSS/p53+ represented 22.7% of the cases (*n* = 22).

Clinicopathological features related to the ACRG subtypes are summarized in Table 2. The MSS/EMT tumors were more common in younger patients (mean age, 55 years) and exhibited mostly diffuse histology by Lauren classification (58.6%).

The gastrectomy history was reported only in MSS/EMT (13.8%) and MSS/p53+ (13.6%) subtypes. In our cohort, 6/97 (6.2%) cases were EBV+. The presence of EBV infection was restricted to MSS cases (3.4% in MSS/EMT, 6.1% in MSS/p53-, and 13.6% in MSS/p53+). HER2 positivity was seen in 1/13 (7.7%) of the MSI, 2/29 (6.9%) of the MSS/EMT, 3/33 (9.1%) of the MSS/p53-, and 3/22 (13.6%) of the MSS/p53+ tumors. PD-L1 positivity was seen more frequently in the MSI subtype (50%). Among intestinal tumors (*n* = 65), 27/65 (41.5%) were MSS/p53-, 15/65 (23.1%) were MSS/p53+, 12/65 (18.5%) were MSS/EMT, and 11/65 (16.9%) were MSI. Among diffuse-mixed tumors (*n* = 32), 17/32 (53.1%) were MSS/EMT, 7/32 (21.9%) were MSS/p53+, 6/32 (18.7) were MSS/p53-, and 2/32 (6.3%) were MSI.

### 3.4. Integrated Classification

The cases were categorized into five subtypes based on the results of EBV, MSI, E-cadherin, and p53 analyses using the stepwise classification algorithm as suggested by previous studies (Figure 1(c)) [7, 8, 10, 21]. Among 97 gastric cancers, EBV positivity was observed in 6 cases (6.2%), MSI in 13 cases (13.4%), E-cadherin aberrant expression in 28 cases (28.9%), p53 aberrant expression in 31 cases (31.9%), and p53 normal expression in 19 cases (19.6%).

The clinicopathological characteristics of patients according to the integrated classification subtypes is summarized in Table 3. In the EBV subtype, 33% of patients had a previous gastric ulcer history, 66% had poorly differentiated tumors, and 100% were males. MSI patients had increased PD-L1 expression (50%) and Lauren intestinal tumors' predominance (84.6%).

Patients in the E-cadherin subtype were diagnosed at a younger age and had a predominance of diffuse/mixed tumors (60.7%). The lack of PD-L1 expression characterized this subtype. p53 aberrant patients had well/moderate differentiated tumors (83.9%) and a history of tobacco smoking (32.3%). Gastric cancers with p53 normal expression were more frequent in elderly patients (mean age, 65 years) and Lauren intestinal tumors (73.7%). Three of the nine (33.3%) HER2+ cases were found in this subtype. Among intestinal tumors (*n* = 65), 26/65 (40%) showed p53 aberrant expression, 14/65 (21.5%) exhibited p53 normal expression, 11/65 (16.9%) were MSI, 11/65 (16.9%) showed E-cadherin aberrant expression, and 3/65 (4.6%) were EBV. Among diffuse-mixed tumors (*n* = 32), E-cadherin aberrant expression was reported in 17/32 (53.1%), followed by p53 aberrant expression in 5/32 (15.6%), p53 normal expression in 5/32 (15.6%), EBV infection in 3/32 (9.4%), and MSI phenotype in 2/32 (6.2%).

### 3.5. Survival Analysis

Patients were followed up from the initial diagnosis date until death, loss to follow-up, or study cut-off date (30 September 2020). From the initial 97 gastric cancer patients, follow-up data were available for 79 (81.4%) patients, whereas 18 (18.6%) patients were lost to follow-up. The median follow-up time was 17 months (range: 0–78 months). The median OS time was 11 months (95% CI; 7.4-14.7) with a 5-year survival rate of 7.2%.

**Figure 3:**
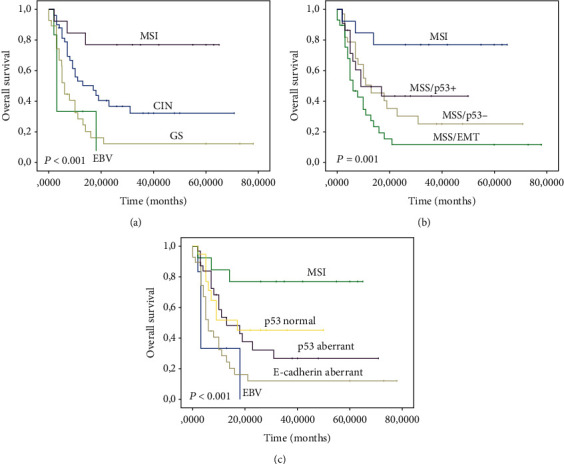
Associations between overall survival and molecular subtypes: (a) TCGA classification, (b) ACRG classification, and (c) integrated classification.

According to TCGA classification, Kaplan-Meier survival curves showed that the MSI subtype had the best prognosis, followed by CIN and GS, whereas the EBV+ subtype exhibited the worst prognosis (log-rank test, *P* < 0.001) (Figure 3(a)). Our findings showed that the ACRG subtypes also correlated with patient OS. The worst OS was seen among MSS/EMT tumors, followed by MSS/p53-, MSS/p53+, and MSI tumors (log-rank test, *P* = 0.001) (Figure 3(b)). Furthermore, the OS of the integrated classification subtypes was analyzed.

The best prognosis was observed in the MSI subtype. In contrast, the EBV subtype displayed the worst prognosis. Patients in p53 normal, p53 aberrant, and E-cadherin aberrant subtypes had the intermediate prognosis (log-rank test, *P* < 0.001) (Figure 3(c)).

Univariate analysis indicated that diffuse/mixed type of Lauren classification, history of gastrectomy, history of gastric ulcer, TCGA classification (EBV vs. MSI, GS, and CIN), ACRG classification (MSS/EMT vs. MSI, MSS/p53-, and MSS/p53+), and integrated classification (EBV vs. MSI, E-cadherin aberrant, p53 aberrant, and p53 normal) were associated with poor prognosis (*P* < 0.05) in our population (Table 4). Multivariate analysis showed that history of gastric ulcer (HR = 4.45; 95% CI, 1.13–17.45; *P* = 0.032) and TCGA subtypes (HR = 7.04; 95% CI, 1.52–32.58; *P* = 0.013) were independent prognostic factors for gastric cancer patients (Table 5).

## 4. Discussion/Conclusion

In the present study, we categorized, for the first time, Moroccan gastric cancers into molecular subtypes using commercially accessible biomarkers and techniques available in routine diagnostic practice. We investigated the associations between gastric cancer molecular subtypes, clinicopathological features, and patient's overall survival. The results showed that diffuse/mixed type of Lauren classification, history of gastrectomy, history of gastric ulcer, TCGA classification (EBV vs. MSI, GS, and CIN), ACRG classification (MSS/EMT vs. MSI, MSS/P53-, and MSS/P53+), and integrated classification (EBV vs. MSI, E-cadherin aberrant, P53 aberrant, and P53 normal) were associated with poor prognosis in our population.

The EBV subtype was reported both in TCGA and integrated gastric cancer classifications. The prevalence of EBV-positive gastric cancers varies widely worldwide (ranging from 0% to 23.6%), with an average rate of approximately 10% [22, 23].

A study conducted on 287 Moroccan gastric cancer patients reported an EBV positivity rate of 28% [24], which is very high compared to the rate (6%) found in our study. The authors detected EBV infection using PCR, which would also detect EBV in surrounding infected lymphocytes, not from tumor cells, resulting in false-positive results [22]. Our study used the gold standard EBER-ISH method for the precise detection of EBV infection [14]. As previously reported in several studies, we noticed a male predominance in the EBV subtype [21, 25–28]. Unlike other studies that reported an association between EBV positivity and better prognosis [29, 30], we found that patients with EBV-positive tumors had the worst prognosis. This discrepancy is probably due to the small sample size (97 patients).

The MSI subtype was reported in all three classifications (TCGA, ACRG, and integrated classification). Thirteen percent (13%) of our samples were MSI, a frequency included within the range published in the literature (8.2-37%) [31]. Several studies reported the importance of MSI status in predicting the response of solid tumors to anti-PD1/PD-L1 immunotherapy [32–35]. The MSI subtype had the best overall survival across all molecular classifications (TCGA, ACRG, and integrated classification). Similar results were reported in other studies [6, 10, 36]. The improved survival of patients with MSI gastric cancer could be explained by the significant T cell infiltration in these tumors. Indeed, MSI+ tumors are characterized by frame-shift mutations, generating abnormal peptides that can be presented to cytotoxic T lymphocytes [37, 38].

Both TCGA and ACRG studies reported a distinctive subtype characterized by the low cell adhesion and the fewest number of mutations, named GS and MSS/EMT, respectively [5, 6]. As expected, the GS and MSS/EMT subtypes shared similarities with the E-cadherin aberrant subtype of the integrated classification. In our cohort, the GS, MSS/EMT, and E-cadherin aberrant subtypes presented the Lauren diffuse histology enrichment and tended to be diagnosed at a younger age.

Besides, the GS, MSS/EMT, and E-cadherin aberrant subtypes had a poor prognosis. These results are consistent with those reported in previous studies [5–7, 36, 39].

Among TCGA subtypes, the CIN subtype was the largest in our cohort, with 51.5% of the cases. This group was characterized by the high frequency of aberrant p53 expression and was found to be enriched in Lauren's intestinal tumors, corresponding to the MSS/p53- (ACRG classification) and p53 aberrant subtypes (integrated classification). This is similar to the results reported in several studies [5, 36]. In line with previous studies, our data showed that CIN tumors are enriched for HER2 overexpression. Therefore, a subset of these patients could be eligible for trastuzumab therapy [40].

Our findings showed that the TCGA classification could better predict the prognosis of patients with gastric cancer compared to ARCG and integrated classifications. Indeed, the TCGA classification and history of gastric ulcer were independent prognostic factors for overall survival.

The major limitation of the present study is the relatively small sample size; only 97 GC patients from a single institution have been included. In addition, some patients were lost to follow-up. Despite these limitations, the strength in the approach lies in the clinical feasibility and its value in predicting the prognosis of gastric cancer patients.

In conclusion, we proposed a reproducible and affordable gastric cancer subtyping algorithms that can reproduce the recently recognized TCGA, ACRG, and integrated gastric cancer classifications, using techniques available in routine diagnosis. Our results showed that these simplified classifications could be employed not only for molecular classification but also for predicting gastric cancer patients' prognosis.

## Figures and Tables

**Table 1:** Clinicopathological features related to the TCGA subtypes.

Variable	*N* (%)	EBV+ (*n* = 6)	MSI^∗^ (*n* = 13)	GS (*n* = 28)	CIN (*n* = 50)	*P* value
Gender						0.094
-Male	59 (60.8%)	6 (100)	7 (53.8)	16 (57.1)	30 (60)	
-Female	38 (39.2%)	0 (0)	6 (46.2)	12 (42.9)	20 (40)	
Mean age at diagnosis (range)	59 (28-85)	61 (46-77)	59 (36-85)	56 (28-76)	62 (39-82)	0.275
Age at diagnosis						0.424
-≥50 years	80 (82.5)	5 (83.3)	9 (69.2)	22 (78.6)	44 (88)	
-<50 years	17 (17.5)	1 (16.7)	4 (30.8)	6 (21.4)	6 (12)	
History of gastrectomy						0.374
-Yes	7 (7.2)	1 (16.7)	0 (0)	3 (10.7)	3 (6)	
-No	90 (92.8)	5 (83.3)	13 (100)	25 (89.3)	47 (94)	
History of gastric ulcer						0.025
-Yes	3 (3.1)	2 (33.3)	0 (0)	0 (0)	1 (2)	
-No	94 (96.9)	4 (66.7)	13 (100)	28 (100)	49 (98)	
Tobacco smoking						0.161
-Yes	25 (25.8)	3 (50)	1 (7.7)	6 (21.4)	15 (30)	
-No	72 (74.2)	3 (50)	12 (92.3)	22 (78.6)	35 (70)	
Tumor location						0.789
-Cardia	31 (31.9)	2 (33.3)	4 (30.8)	11 (39.3)	14 (28)	
-Noncardia	66 (68.1)	4 (66.7)	9 (69.2)	17 (60.7)	36 (72)	
Tumor cells differentiation						0.081
-Well/moderate	62 (63.9)	2 (33.3)	7 (53.8)	15 (53.6)	38 (76)	
-Poor	35 (36.1)	4 (66.7)	6 (46.2)	13 (46.4)	12 (24)	
Lauren classification						0.001
-Intestinal	65 (67.1)	3 (50)	11 (84.6)	11 (39.3)	40 (80)	
-Diffuse/mixed	32 (32.9)	3 (50)	2 (15.4)	17 (60.7)	10 (20)	
HER2 immunostaining						0.906
-Positive	9 (9.3)	1 (16.7)	1 (7.7)	2 (7.1)	5 (10)	
-Negative	88 (90.7)	5 (83.3)	12 (92.3)	26 (92.9)	45 (90)	
p53 immunostaining						0.134
-Aberrant	49 (50.5)	2 (33.3)	5 (38.5)	11 (39.3)	31 (62)	
-Normal	48 (49.5)	4 (66.7)	8 (61.5)	17 (60.7)	19 (38)	
PD-L1 immunostaining	*n* = 38	*n* = 3	*n* = 6	*n* = 10	*n* = 19	0.177
-Positive	6 (15.8)	1 (33.3)	3 (50)	0 (0)	2 (10.5)	
-Negative	32 (84.2)	2 (66.7)	3 (50)	10 (100)	17 (89.5)	

TCGA: The Cancer Genome Atlas; EBV: Epstein-Barr virus; MSI: microsatellite instability; GS: genome stable; CIN: chromosomal instability; ^∗^previously reported [13].

**Table 2:** Clinicopathological features related to the ACRG subtypes.

Variable	*N* (%)	MSI^∗^ (*n* = 13)	MSS/EMT (*n* = 29)	MSS/p53- (*n* = 33)	MSS/p53+ (*n* = 22)	*P* value
Gender						0.917
-Male	59 (60.8%)	7 (53.8)	17 (58.6)	21 (63.6)	14 (63.6)	
-Female	38 (39.2%)	6 (46.2)	12 (41.4)	12 (36.4)	8 (36.4)	
Mean age at diagnosis (range)	59 (28-85)	59 (36-85)	55 (28-76)	60 (39-80)	64 (39-82)	0.101
Age at diagnosis						0.245
-≥50 years	80 (82.5)	9 (69.2)	22 (75.9)	29 (87.9)	20 (90.9)	
-<50 years	17 (17.5)	4 (30.8)	7 (24.1)	4 (12.1)	2 (9.1)	
History of gastrectomy						0.023
-Yes	7 (7.2)	0 (0)	4 (13.8)	0 (0)	3 (13.6)	
-No	90 (92.8)	13 (100)	25 (86.2)	33 (100)	19 (86.4)	
History of gastric ulcer						0.222
-Yes	3 (3.1)	0 (0)	0 (0)	1 (3)	2 (9.1)	
-No	94 (96.9)	13 (100)	29 (100)	32 (97)	20 (90.9)	
Tobacco smoking						0.207
-Yes	25 (25.8)	1 (7.7)	6 (20.7)	11 (33.3)	7 (31.8)	
-No	72 (74.2)	12 (92.3)	23 (79.3)	22 (66.7)	15 (68.2)	
Tumor location						0.156
-Cardia	31 (31.9)	4 (30.8)	11 (37.9)	13 (39.4)	3 (13.6)	
-Noncardia	66 (68.1)	9 (69.2)	18 (62.1)	20 (60.6)	19 (86.4)	
Tumor cells differentiation						0.079
-Well/moderate	62 (63.9)	7 (53.8)	16 (55.2)	27 (81.8)	12 (54.5)	
-Poor	35 (36.1)	6 (46.2)	13 (44.8)	6 (18.2)	10 (45.5)	
Lauren classification						0.003
-Intestinal	65 (67.1)	11 (84.6)	12 (41.4)	27 (81.8)	15 (68.2)	
-Diffuse/mixed	32 (32.9)	2 (15.4)	17 (58.6)	6 (18.2)	7 (31.8)	
HER2 immunostaining						0.874
-Positive	9 (9.3)	1 (7.7)	2 (6.9)	3 (9.1)	3 (13.6)	
-Negative	88 (90.7)	12 (92.3)	27 (93.1)	30 (90.9)	19 (86.4)	
EBV						0.296
-Positive	6 (6.2)	0 (0)	1 (3.4)	2 (6.1)	3 (13.6)	
-Negative	91 (93.8)	13 (100)	28 (96.6)	31 (93.9)	19 (86.4)	
PD-L1 immunostaining	*n* = 38	*n* = 6	*n* = 11	*n* = 11	*n* = 10	0.418
-Positive	6 (15.8)	3 (50)	1 (9.1)	1 (9.1)	1 (10)	
-Negative	32 (84.2)	3 (50)	10 (90.9)	10 (90.9)	9 (90)	

MSI: microsatellite instability; MSS/EMT: microsatellite stable with epithelial-to-mesenchymal transition; MSS/p53-: microsatellite stable with inactive p53; MSS/p53+: microsatellite stable with active p53; ^∗^previously reported [13].

**Table 3:** Clinicopathological features related to the integrated classification subtypes.

Variable	*N* (%)	EBV+ (*n* = 6)	MSI^∗^ (*n* = 13)	E-cadherin aberrant (*n* = 28)	p53 aberrant (*n* = 31)	p53 normal (*n* = 19)	*P* value
Gender							0.168
-Male	59 (60.8%)	6 (100)	7 (53.8)	16 (57.1)	19 (61.3)	11 (57.9)	
-Female	38 (39.2%)	0 (0)	6 (46.2)	12 (42.9)	12 (38.7)	8 (42.1)	
Mean age at diagnosis (range)	59 (28-85)	61 (46-77)	59 (36-85)	56 (28-76)	60 (39-80)	65 (39-82)	0.218
Age at diagnosis							0.581
-≥50 years	80 (82.5)	5 (83.3)	9 (69.2)	22 (78.6)	27 (87.1)	17 (89.5)	
-<50 years	17 (17.5)	1 (16.7)	4 (30.8)	6 (21.4)	4 (12.9)	2 (10.5)	
History of gastrectomy							0.055
-Yes	7 (7.2)	1 (16.7)	0 (0)	3 (10.7)	0 (0)	3 (15.8)	
-No	90 (92.8)	5 (83.3)	13 (100)	25 (89.3)	31 (100)	16 (84.2)	
History of gastric ulcer							0.023
-Yes	3 (3.1)	2 (33.3)	0 (0)	0 (0)	0 (0)	1 (5.3)	
-No	94 (96.9)	4 (66.7)	13 (100)	28 (0)	31 (100)	18 (94.7)	
Tobacco smoking							0.252
-Yes	25 (25.8)	3 (50)	1 (7.7)	6 (21.4)	10 (32.3)	5 (26.3)	
-No	72 (74.2)	3 (50)	12 (92.3)	22 (78.6)	21 (67.7)	14 (73.7)	
Tumor location							0.186
-Cardia	31 (31.9)	2 (33.3)	4 (30.8)	11 (39.3)	12 (38.7)	2 (10.5)	
-Noncardia	66 (68.1)	4 (66.7)	9 (69.2)	17 (60.7)	19 (61.3)	17 (89.5)	
Tumor cells differentiation							0.051
-Well/moderate	62 (63.9)	2 (33.3)	7 (53.8)	15 (53.6)	26 (83.9)	12 (63.2)	
-Poor	35 (36.1)	4 (66.7)	6 (46.2)	13 (46.4)	5 (16.1)	7 (36.8)	
Lauren classification							0.002
-Intestinal	65 (67.1)	3 (50)	11 (84.6)	11 (39.3)	26 (83.9)	14 (73.7)	
-Diffuse/mixed	32 (32.9)	3 (50)	2 (15.4)	17 (60.7)	5 (16.1)	5 (26.3)	
HER2 immunostaining							0.789
-Positive	9 (9.3)	1 (16.7)	1 (7.7)	2 (7.1)	2 (6.5)	3 (15.8)	
-Negative	88 (90.7)	5 (83.3)	12 (92.3)	26 (92.9)	29 (93.5)	16 (84.2)	
PD-L1 immunostaining	*n* = 38	*n* = 3	*n* = 6	*n* = 10	*n* = 11	*n* = 8	0.324
-Positive	6 (15.8)	1 (33.3)	3 (50)	0 (0)	1 (9.1)	1 (12.5)	
-Negative	32 (84.2)	2 (66.7)	3 (50)	10 (100)	10 (90.9)	7 (87.5)	

EBV: Epstein-Barr virus; MSI: microsatellite instability; ^∗^previously reported [13].

**Table 4:** Univariate analysis of overall survival.

Variable	Mean OS months (95% CI)	*P* value
Gender		0.127
-Male	22.73 (16.09-29.38)	
-Female	39.38 (26.62-52.13)	
Age at initial diagnosis		0.162
->50 years	28.74 (20.92-36.55)	
-≤50 years	34.58 (21.92-47.24)	
Tumor location		0.410
-Cardia	26.27 (14.77-37.76)	
-Noncardia	32.03 (23.64-40.43)	
Tumor cells differentiation		0.066
-Poor	26.49 (14.91-38.06)	
-Well/moderate	32.08 (23.86-40.33)	
Lauren classification		0.014
-Intestinal	33.09 (24.95-41.23)	
-Diffuse/mixed	22.19 (11.04-33.34)	
History of gastrectomy		0.030
-Absent	33.51 (25.82-41.19)	
-Present	8.59 (2.98-14.19)	
History of gastric ulcer		0.003
-Absent	32.26 (24.87-39.64)	
-Present	3.67 (2.34-5.00)	
Smoking		0.674
-No	32.04 (23.67-40.41)	
-Yes	23.21 (12.69-33.73)	
HER2 immunostaining		0.956
-Positive	18.35 (7.15-29.56)	
-Negative	31.01 (23.48-38.55)	
PD-L1 immunostaining		0.658
-Positive	16.39 (5.67-27.11)	
-Negative	25.71 (15.48-35.93)	
TCGA classification		<0.001
-EBV	7.85 (1.39-14.30)	
-MSI	51.78 (38.59-64.98)	
-GS	15.65 (6.41-24.89)	
-CIN	30.26 (20.71-39.82)	
ACRG classification		0.001
-MSI	51.78 (38.59-64.98)	
-MSS/EMT	15.69 (6.82-24.56)	
-MSS/P53-	26.24 (15.27-37.21)	
-MSS/P53+	25.51 (15.74-35.28)	
Integrated classification		<0.001
-EBV	7.85 (1.39-14.30)	
-MSI	51.78 (38.59-64.98)	
-E-cadherin aberrant	15.65 (6.41-24.89)	
-P53 aberrant	27.74 (16.26-39.22)	
-P53 normal	26.69 (16.26-37.13)	

**Table 5:** Multivariate analysis of the prognostic factors for patients with gastric can.

Variable	Hazard ratio	95% CI	*P* value
TCGA classification (EBV vs. MSI, GS, and CIN)	7.04	1.52–32.58	0.013
History of gastric ulcer (presence vs. absence)	4.45	1.13–17.45	0.032

GC: gastric cancer; TCGA: The Cancer Genome Atlas research; MSI: microsatellite instability; CIN: chromosomal instability; EBV: Epstein-Barr virus; GS: genome stable; CI: confidence interval.

## Data Availability

The processed data are available from the corresponding author upon reasonable request.

## Supplementary Materials

Supplementary Materials Figure 2 (supplementary material): immunohistochemical analysis: (G) HER2 negative, (H) HER2 positive, (I) PD-L1 negative, and (J) PD-L1 positive (magnification ×400).
