# Supplementary material for: Reproduction of the Cancer Genome Atlas (TCGA) and Asian Cancer Research Group (ACRG) Gastric Cancer Molecular Classifications and Their Association with Clinicopathological Characteristics and Overall Survival in Moroccan Patients
Source: Dis Markers. 2021 Jul 28;2021:9980410. doi: 10.1155/2021/9980410 (PMC8342151; doi:10.1155/2021/9980410)
Supplement: Supplementary Materials — Figure 2 (supplementary material): immunohistochemical analysis: (G) HER2 negative, (H) HER2 positive, (I) PD-L1 negative, and (J) PD-L1 positive (magnification ×400). [file 9980410.f1.pdf]

Figure 2 (supplementary material)

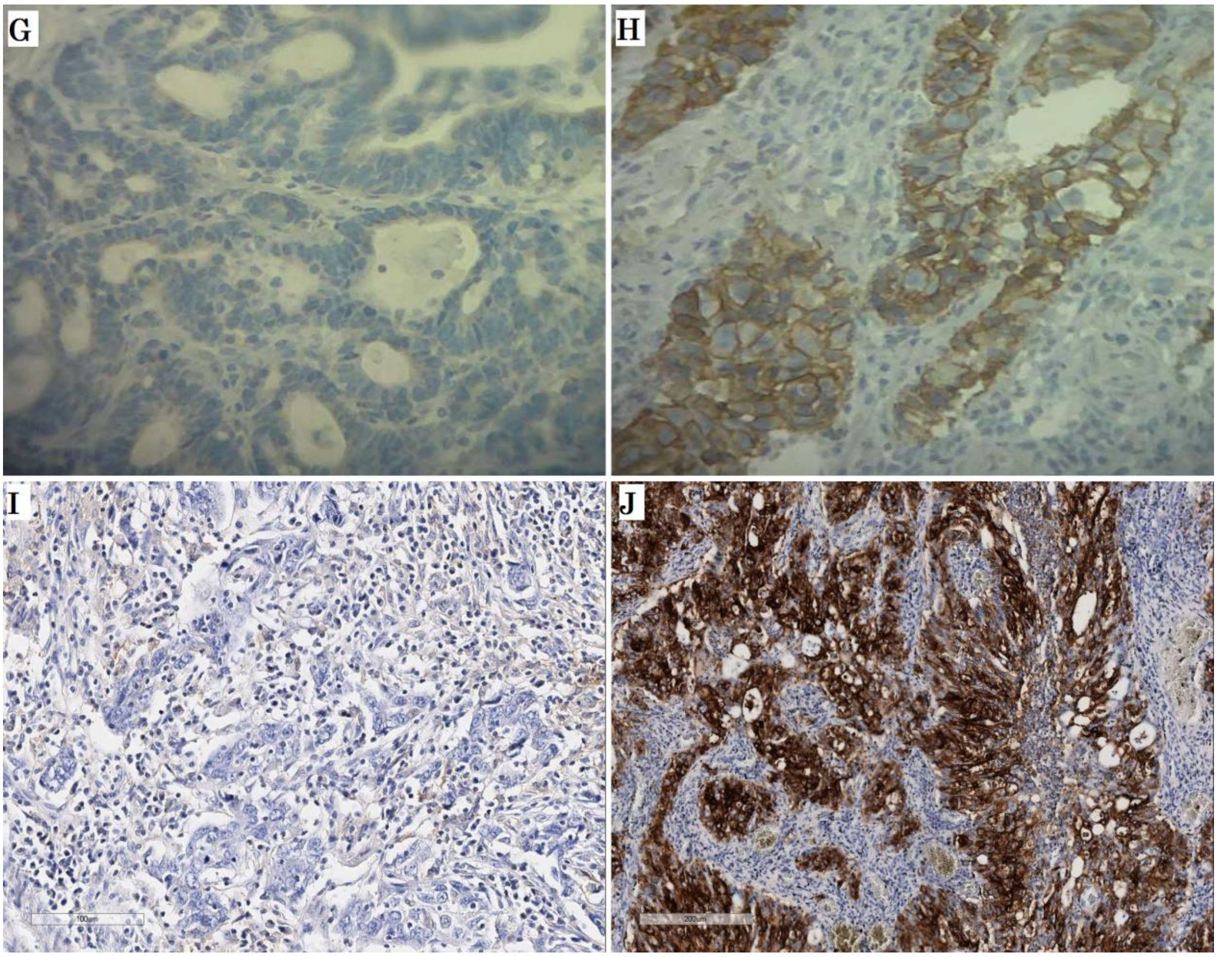

Immunohistochemical analysis: (G) HER2 negative, (H): HER2 positive, (I) PD-L1 negative and (J) PD-L1 positive (magnification x400).
